# Assessing the effectiveness of endoscopic submucosal dissection for hypopharyngeal lesions- a comprehensive evaluation

**DOI:** 10.3389/fonc.2026.1738370

**Published:** 2026-05-29

**Authors:** Changen Liu, Qiang Zhang, Yaohui Wang, Tingsheng Ling

**Affiliations:** 1 Department of Gastroenterology and Hepatology, Central Hospital, Tianjin University /Tianjin Third Central Hospital, Tianjin Institute of Geriatrics, Tianjin Third Central Hospital Branch, Tianjin, China; 2 Digestive Endoscopy Center, Affiliated Hospital of Nanjing University of Chinese Medicine (Jiangsu Province Hospital of Chinese Medicine), Nanjing, China; 3 Pathology Department, Affiliated Hospital of Nanjing University of Chinese Medicine (Jiangsu Province Hospital of Chinese Medicine), Nanjing, China

**Keywords:** endoscopic submucosal dissection, hypopharyngeal carcinoma, safety, superficial lesions, treatment efficacy

## Abstract

**Background:**

Superficial hypopharyngeal carcinoma typically presents with no obvious clinical manifestations but poses significant management challenges due to its unique anatomical location and potential aggressiveness.

**Objective:**

To evaluate the clinical efficacy and safety of endoscopic submucosal dissection (ESD) in the treatment of superficial hypopharyngeal carcinoma.

**Methods:**

We retrospectively analyzed the clinical data of 11 patients with 13 superficial hypopharyngeal carcinomas who underwent ESD at the Gastrointestinal Endoscopy Center of Jiangsu Provincial Hospital of Traditional Chinese Medicine from November 2022 to November 2023.

**Results:**

All 11 patients were male, with 13 lesions located as follows: left pyriform fossa (53.8%), right pyriform fossa (23.1%), posterior hypopharyngeal wall (15.4%), and posterior cricoid region (7.7%). The average lesion diameter was 1.79 cm (range: 0.1–3.9 cm). The procedure took an average of 34 minutes (range: 10–90 minutes). Intraoperatively, subcutaneous cervicothoracic emphysema occurred in 1 patient (7.7%). Postoperatively, 5 patients (38.5%) reported postoperative pain, and 1 patient (7.7%) developed postoperative fever. During follow-up, choking symptoms were noted in 2 cases (18.2%), and hoarseness occurred in 4 cases (36.4%). The average length of hospitalization was 7.3 days (range: 5–10 days). Postoperative pathology revealed intraepithelial high-grade neoplasia in 46.2% of lesions, carcinoma *in situ* in 7.7%, and invasive carcinoma in 46.2%. One case of squamous cell carcinoma had positive vertical margins, requiring additional radiotherapy and chemotherapy, while the remaining lesions had negative peripheral and basal margins. The mean follow-up period was 17 months (range: 9–25 months), with no recurrence, metastasis, or esophageal inlet stenosis observed in any patient.

**Conclusion:**

ESD is a safe and effective treatment for superficial hypopharyngeal carcinoma and can be considered a viable option for the minimally invasive treatment of this condition.

## Introduction

According to the International Agency for Research on Cancer (IARC), there were approximately 21,000 new cases of hypopharyngeal carcinoma in China in 2020, accounting for 25% of the global total. The age−standardized incidence rate (ASIR) was 1.00 per 100,000 population, and the mortality rate was 0.46 per 100,000 population (1). Although superficial hypopharyngeal carcinoma is relatively rare, it presents a significant clinical challenge due to its unique anatomical location and potential for invasive progression. Traditional treatments, including surgery, radiotherapy, and chemotherapy, each have their own limitations and complications. Endoscopic submucosal dissection (ESD) has emerged as a minimally invasive alternative for treating superficial hypopharyngeal carcinoma, offering precise tumor removal while preserving organ function. Originally developed for gastrointestinal lesions, ESD has shown promising results in the treatment of superficial hypopharyngeal cancer. However, comprehensive data on its efficacy and safety remain limited. This study aims to analyze and summarize the clinical data of 11 patients with superficial hypopharyngeal lesions treated with ESD to evaluate its clinical efficacy and safety for superficial hypopharyngeal cancer.

## Patients and methods

### Study participants

Eleven male patients with superficial hypopharyngeal carcinoma who underwent ESD at the Gastrointestinal Endoscopy Center of Affiliated Hospital of Nanjing University of Chinese Medicine from November 2022 to November 2023 were retrospectively included in this study. The patients were aged 50–77 years (mean age: 62.9 ± 6.9 years). All participants were asymptomatic and had a history of alcohol consumption and smoking for more than 10 years. Among the patients, 7 (63.6%) had concurrent esophageal tumors, 3 (27.3%) had heterochronous esophageal tumors, and 2 (18.2%) had concurrent gastric tumors. All patients were diagnosed by biopsy before surgery, and imaging evaluations revealed no obvious cervical lymph node or distant metastasis prior to the procedure (**Table 1**).

**Table 1:** Baseline characteristics of patients and lesions.

Characteristic	Case
Patients	11
Average age (y)	62.9 ± 6.9
Gender	
Male	11 (100.0)
Female	0 (0.0)
More than 10 years of smoking history	
Yes	11 (100.0)
No	0 (0.0)
More than 10 years of alcohol consumption	
Yes	11 (100.0)
No	0 (0.0)
History of tumor	
Esophageal tumors	10 (90.9)
Simultaneous	7 (63.6)
Heterochronic	3 (27.3)
Gastric tumors	2 (18.2)
Simultaneous	2 (18.2)
Heterochronic	0 (0.0)

### ESD procedures

#### Endoscopes and instruments

Electronic Gastroscope System (Olympus EVIS 290); Transparent Cap (Olympus MH-597); ICC-200 High Frequency Electrode (ERBE, Germany); Dual Knife (Olympus, Japan); Thermal Biopsy Forceps (Olympus FD-410LR); Hemostatic Clamps, etc.

#### Procedure

Under general anesthesia with tracheal intubation, the hypopharyngeal lesion was localized using endoscopy. Before surgery, the extent of the lesion was assessed by staining or magnification to visualize the glandular duct openings and blood vessel patterns. Circumferential marking was made 0.5 cm lateral to the lesion’s edge. Repeated injections of 0.01% epinephrine and melphalan solution were administered at the lateral edges to elevate the lesion. After localized window opening, a Dual knife was used to make an incision along the lesion margin. The submucosal layer beneath the lesion was carefully dissected using the Dual knife, with additional submucosal injections to maintain the plane of dissection. After excision, bleeding was controlled using thermal biopsy forceps via electrocoagulation. The resected lesion was retracted with the help of dental floss. Postoperative microscopic examination confirmed no residual tumor at the excision margins.

### Specimen processing

After excising the lesion, the specimen was spread and fixed on a specimen plate, then stained with iodine solution to confirm complete excision. The specimen was soaked in 10% neutral formalin for 24 hours and then sliced into strips with a width of 2.0-3.0 mm. After embedding in wax, the tissue was sliced and stained. The presence of cancerous cells at the vertical and horizontal margins, as well as any invasion into blood vessels, lymphatic vessels, or nerves, was evaluated.

### Postoperative management and follow-up

**Figure 1:**
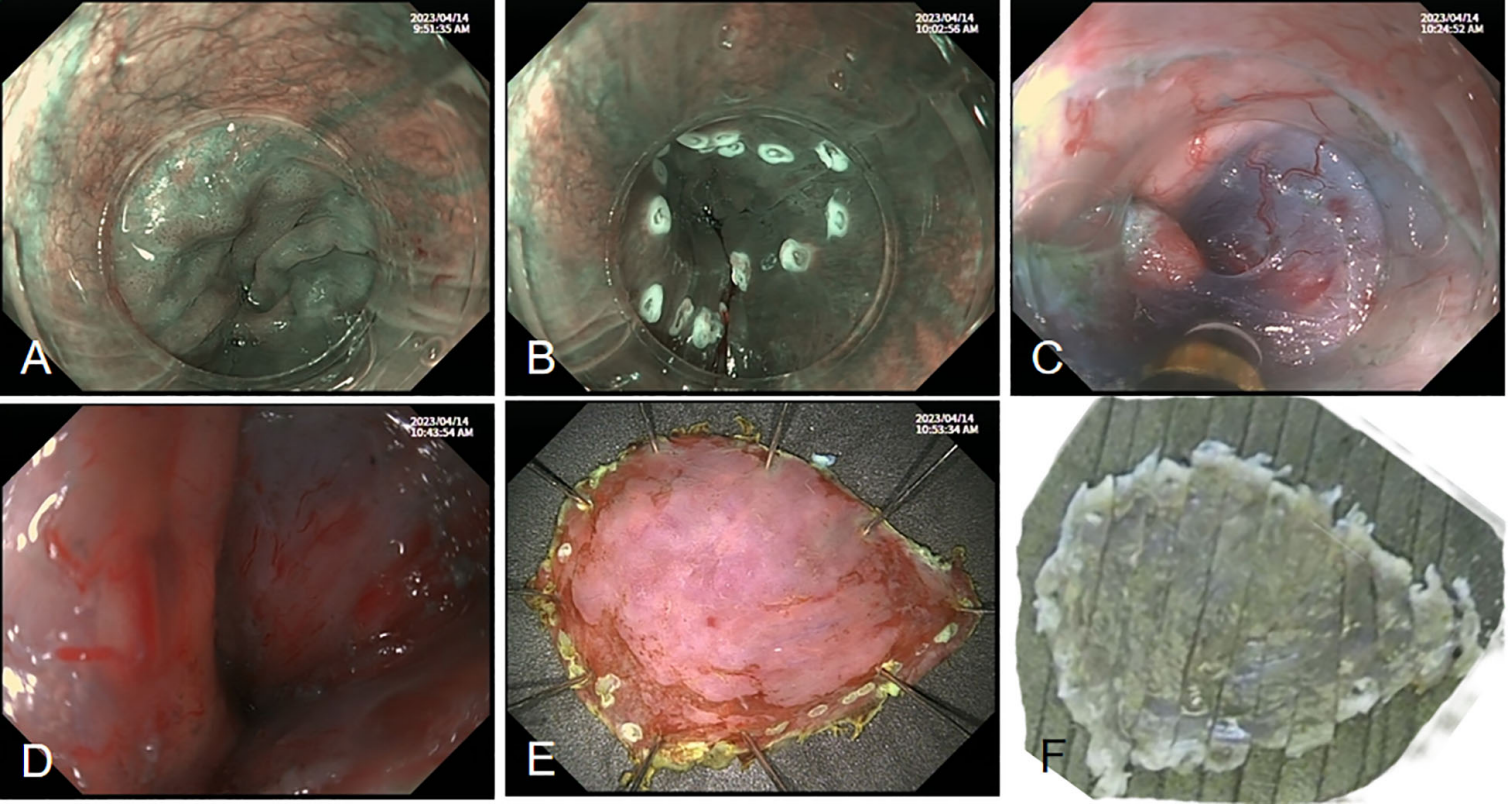
Procedure of ESD for hypopharyngeal tumor. **(A)** Tone change observed under Narrow Band Imaging (NBI); **(B)** Electrocoagulation used to mark the margin of the lesion. **(C)** Intraoperative titanium clip and dental floss traction applied, followed by pre-incision of the outer mucosa along the marked points. **(D)** Post-Endoscopic Submucosal Dissection defect wound. **(E)** Submucosal dissection of the lesion. **(F)** Postoperative pathological resection image.

**Figure 2:**
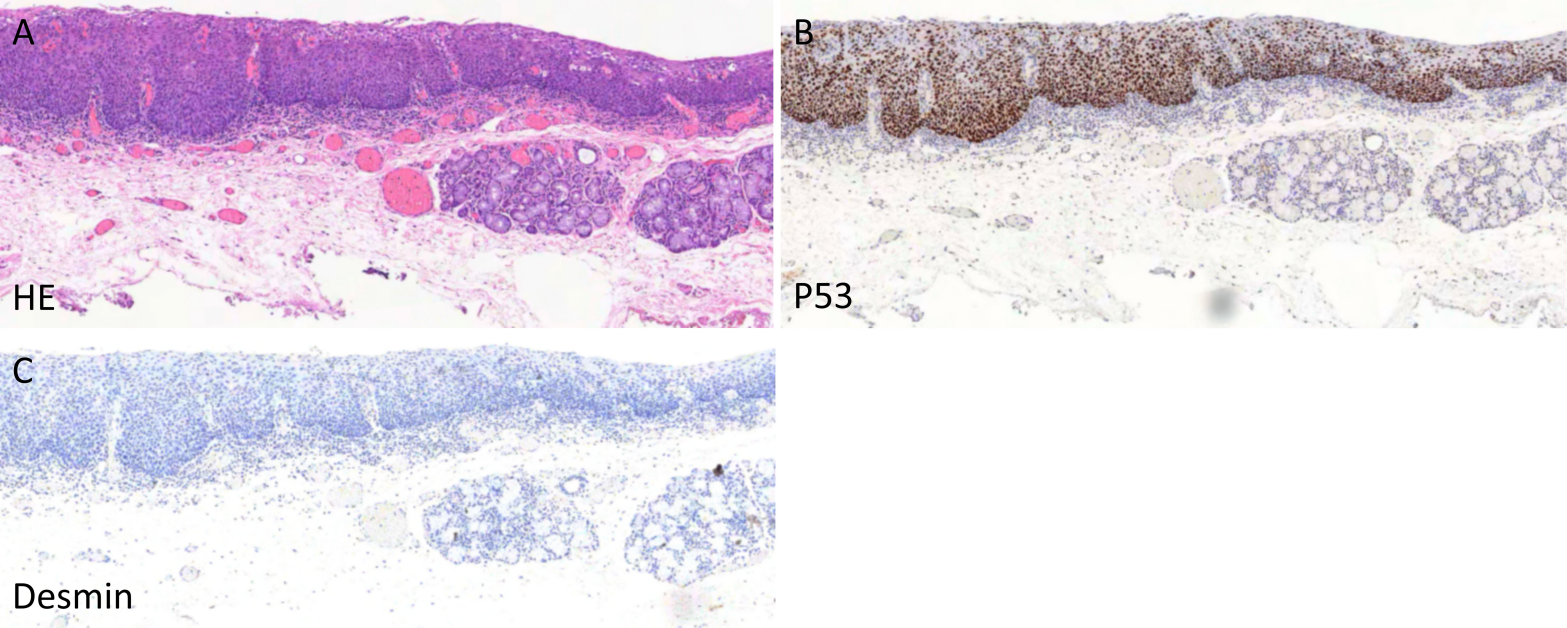
Postoperative specimen handling and pathological evaluation: **(A)** Section staining at HEx40 magnification of some of the sampling sites. **(B)** P53 shows positive. **(C)** Desmin shows negative, that suggests the absence of the muscularis mucosae in the mucosa of the hypopharynx.

Patients were kept on nil per os (NPO) for 24–48 hours post-surgery to prevent aspiration and were given appropriate supportive care, including acid suppression and rehydration. Gastroscopy was performed 3 months after the operation, followed by follow-up gastroscopy every 6 months to 1 year. Enhanced CT scans and chest radiographs were performed annually to monitor for lymph node involvement or distant metastases (**Figures 1**, **2**).

### Data analysis

Normally distributed quantitative data were expressed as ( $\bar{\text{x}}\pm \text{s}$), obviously skewed quantitative data were expressed as M (range), and categorical data were expressed as frequency (%).

## Results

### Therapeutic outcomes

A total of 13 lesions from 11 patients were analyzed. The lesions were located in the left pyriform fossa in 7 cases (53.8%), the right pyriform fossa in 3 cases (23.1%), the posterior wall of the hypopharynx in 2 cases (15.4%), and the posterior region of the cricoid in 1 case (7.7%). The average lesion diameter was 1.79 cm (range 0.1-3.9 cm), with 6 lesions (46.2%) having a diameter of ≤2 cm and 7 lesions (53.8%) having a diameter >2 cm.

All patients successfully completed the ESD procedure, with an average operation duration of 34 minutes (range 10–90 minutes). No intraoperative bleeding was observed. Subcutaneous cervico-thoracic emphysema occurred in 1 case (7.7%), which resolved spontaneously following high-concentration oxygen inhalation. In 1 case (7.7%), limited operating space was observed due to osseous structure and tracheal intubation. The lesion was removed using a snare, followed by electrocoagulation and further resection at the anal side resection margin.

Postoperatively, all patients experienced varying degrees of foreign body sensation in the pharynx. Five patients (38.5%) reported postoperative pain, which resolved without special treatment. No delayed hemorrhage was observed. Postoperative fever was found in 1 patient (7.7%), which improved with symptomatic treatment. The average length of hospitalization was 7.3 days (range 5–10 days) (**Table 2**).

**Table 2:** Treatment progress.

Characteristic	Case
Median lesion diameter (mm)	20
Median operating time (min)	35
Curative resection rate	7 (53.8)
Cutting edge	
Horizontal margin positive	6 (46.2)
Vertical margin positive	1 (7.7)
Ulcers	1 (7.7)
Location	
Pyriform crypt	10 (76.9)
Left	7 (53.8)
Right	3 (23.1)
Posterior pharyngeal wall	2 (15.4)
Post-cricoid region	1 (7.7)
Tumor diameter(mm)	
≤10	4 (30.8)
11-20	3 (23.1)
21-30	5 (38.5)
>30	1 (7.7)

### Postoperative pathology and follow-up

#### Postoperative pathology

Six cases (46.2%) were diagnosed with high-grade intraepithelial neoplasia, 1 case (7.7%) with carcinoma *in situ*, and 6 cases (46.2%) with invasive carcinoma. Among the invasive carcinoma cases, there were 2 cases each of poorly differentiated, moderately differentiated, and highly differentiated squamous cell carcinoma. One case (7.7%) exhibited ulcerative infiltration, and 1 case had positive vertical margins, for which 32 additional radiotherapy sessions and 8 chemotherapy treatments were administered. During the 16-month follow-up, no recurrence or metastasis was observed in this patient (**Table 3**).

**Table 3:** Histological result.

Physiology	Case
High-grade intraepithelial neoplasia/Carcinoma in situ	6 (53.9)
Highly differentiated squamous cell carcinoma	2 (15.4)
Moderately Differentiated Squamous Cell Carcinoma	2 (15.4)
Low-differentiated squamous cell carcinoma	2 (15.4)

#### Follow-up

Intraoperatively, subcutaneous cervicothoracic emphysema occurred in 1 patient (7.7%). Postoperatively, 5 patients (38.5%) reported postoperative pain, and 1 patient (7.7%) developed postoperative fever. During follow-up, choking symptoms were noted in 2 cases (18.2%), and hoarseness occurred in 4 cases (36.4%). No residual lesions, recurrence, or metastasis were observed, and there were no fatal cases (**Table 4**).

**Table 4:** Complications of ESD for early hypopharyngeal cancers.

Complications	Case
Yes	5 (38.5)
Subcutaneous cervicothoracic emphysema	1 (7.7)
Postoperative pain	5 (38.5)
postoperative fever	1 (7.7)
Choking symptoms	2 (15.4)
Hoarseness	4 (30.8)
No	8 (61.5)

## Discussion

Hypopharyngeal carcinomas are predominantly squamous cell carcinomas, accounting for 3-5% of head and neck cancers. The pyriform fossa is the most common site (65-85%), followed by the posterior hypopharyngeal wall (10-20%) and posterior cricoid region (5-15%) (2). Most hypopharyngeal cancers are diagnosed at advanced stages (III or IV), resulting in a poor prognosis (3). Risk factors include male sex, smoking, alcohol consumption, and the presence of second primary tumors, especially in the esophagus (4). In this study, 90.9% of patients had concurrent or heterochronous esophageal cancer, and 18.2% had early gastric cancer. This suggests that shared risk factors, such as the squamous epithelium lining both the hypopharynx and esophagus, may increase the likelihood of simultaneous malignancies.

Despite advances in endoscopic techniques such as NBI and magnifying endoscopy, superficial hypopharyngeal carcinomas remain difficult to detect, with detection rates as low as 6.3% (5). Therefore, a combination of white light endoscopy, electronic staining, and targeted screening is essential to improve detection rates.

ESD initially used for gastrointestinal cancers, has not been widely applied to hypopharyngeal carcinomas due to anatomical challenges, such as the narrowed lumen and susceptibility to lymph node metastasis. However, ESD has shown promising results in treating superficial hypopharyngeal cancer, offering high rates of curative and complete resection, which help reduce recurrence risks (6–9). In recent years, the indications for ESD have been continuously refined through research. It is currently universally recognized that ESD is indicated for superficial hypopharyngeal carcinoma, defined as lesions where cancer cells invade the epithelium or subepithelial layer but not the muscularis propria, without evidence of definite lymph node or distant metastasis (stage T1a-T1b). A single-center study published in 2025 demonstrated that ESD is applicable for superficial lesions with a maximum diameter of ≤3 cm, particularly those displaying microvascular features of intraepithelial papillary capillary loop (IPCL) type B1 on NBI combined with magnifying endoscopy. Such lesions can achieve curative resection via ESD, obviating the need for adjuvant chemoradiotherapy (10). In addition, recent case series have shown that for patients with suspected synchronous lymph node metastasis who desire to maintain their quality of life, ESD combined with endoscopic laryngopharyngeal surgery (ELPS) can serve as an alternative strategy. This addresses the limitation of ESD alone in managing lymph node involvement (11).

The indications for EMR are narrower than those for ESD, primarily encompassing small, superficial, and non-invasive lesions (stage T0-T1a). A retrospective study indicated that EMR is preferentially indicated for superficial squamous lesions with a maximum diameter of ≤1 cm and an invasion depth of ≤200μm, without significant fibrosis or vascular invasion (12). Given the difficulty of achieving en bloc resection of large lesions with EMR, its clinical utility is limited for superficial lesions with a diameter >1 cm or irregular morphology, as incomplete resection is associated with an increased risk of recurrence.

The indications for conventional surgical approaches (including open partial laryngopharyngectomy, total laryngectomy, cervical lymph node dissection, as well as transoral laser microsurgery (TLM) and transoral robotic surgery) primarily include middle and advanced hypopharyngeal tumors (stage T2-T4) or cases with definite cervical lymph node metastasis. For patients with a tumor invasion depth >1000μm, lesions of IPCL type B2/B3, or confirmed cervical lymph node metastasis, conventional surgery combined with lymph node dissection remains the first-line treatment, as it effectively reduces the local recurrence rate (13). Additionally, conventional surgery serves as an important salvage therapy for patients with failed endoscopic resection, postoperative recurrence, or contraindications to endoscopic procedures. In our study, the R0 resection rate was 92.3%, which is higher than the pooled results from a review by Kamal F et al. (14), although larger multicenter studies are needed to confirm this finding.

While complete lesion resection is critical, preserving surrounding physiological structures is essential to avoid complications such as bleeding, pharyngeal edema, and vocal cord paralysis. Traditional treatments like surgery and radical radiotherapy often lead to significant functional impairment and long recovery times. In contrast, ESD offers a minimally invasive approach that preserves organ function, reduces postoperative discomfort, and improves quality of life. In our cohort, no intraoperative perforation, delayed bleeding, or serious complications occurred, and residual symptoms such as choking and hoarseness resolved within 1–2 months.

A meta-analysis from Japan (15) found that traction techniques during ESD improve resection clarity, reduce risks, shorten operative time, and promote better postoperative recovery. In our study, floss traction was used in all patients, ensuring refined and well-controlled surgery.

Long-term follow-up studies also demonstrate favorable outcomes with ESD. Muto et al. (16) reported a 5-year survival rate of 71% and a disease-specific survival rate of 97%. Another study showed a 5-year survival rate of 80.7% and a 100% cause-specific survival rate for superficial hypopharyngeal carcinoma treated with ESD (17). Our study also supports these findings: no recurrences were observed after a mean follow-up of 17 months (9–25 months), except for one patient who required additional radiotherapy due to positive vertical margins.

A 2024 Japanese study by Matsuura et al. adopted water pressure-assisted ESD in 21 patients with 21 lesions (mean size 10.0 mm). The mean operation time was 15 min, with 100% en bloc resection and 76.2% R0 resection rates. No recurrence was noted, while 2 patients developed postoperative subcutaneous emphysema, and all patients achieved full recovery (18). Gong et al. performed conventional ESD on 41 patients with 54 lesions (25.7 mm on average) in China in 2022. The average procedure time was 49.1 min, with en bloc and R0 resection rates of 98.2% and 74.5%, respectively. Only one local recurrence and two cases of delayed postoperative hemorrhage were reported, and all patients recovered completely (19). In 2021, Chen et al. treated 27 patients with 30 hypopharyngeal lesions (mean 15.0 mm) using standard ESD. The operative time averaged 36.0 min, achieving 100% en bloc resection and 93.3% R0 resection. One patient suffered from emphysema without recurrence, and all cases had a full recovery (20). Hanaoka’s 2013 Japanese study included 28 patients and 38 small lesions (6.2 mm); the mean procedure time was 74.0 min, with 100% en bloc resection and 81.6% R0 resection. Four cases of local recurrence and four cases of laryngeal edema were observed, and all patients recovered well (21). In 2012, Iizuka et al. applied peroral countertraction-assisted ESD to 93 patients with 140 lesions (23.4 mm), with a mean operative time of 65.3 min. This modified technique yielded 100% en bloc resection and 69.8% R0 resection, with no recurrence or complications and full recovery in all subjects (22). Another 2011 study by Iizuka enrolled 32 patients with 42 lesions (12.5 mm) treated with conventional ESD. The procedure lasted 98.0 min on average, with 98% en bloc resection and 79.0% R0 resection. Two patients had subcutaneous emphysema without recurrence, and all achieved full recovery (23). As the earliest included trial, Shimizu et al. reported a small sample of 4 patients with 4 lesions in 2006. The mean ESD time was 68.8 min, with relatively low en bloc (75%) and R0 (50.0%) resection rates; no recurrence or complications occurred, and all patients recovered fully (24). Overall, ESD and its modified techniques maintain high en bloc resection rates for hypopharyngeal lesions. Postoperative complications are mild and infrequent, with rare local recurrence (**Table 5**).

**Table 5:** Literature review of endoscopic submucosal dissection for hypopharyngeal lesions.

No.	Year	Author	Study location	Number of cases	Number of lesions	Lesion size (mm)	Procedure time (min)	Therapeutic regimen	En bloc resection rate	R0 resection	Recurrence after ESD	Postoperative complication	Clinical outcome
1	2024	Noriko Matsuura	Japan	21	21	10.0	15	water pressure method for ESD	100%	76.2%	No	postoperative subcutaneous emphysema occurred in 2 patients	Full recovery (18)
2	2022	Yu Gong	China	41	54	25.7	49.1	ESD	98.2%	74.5%	local recurrence was observed in 1 patients	delayed postoperative hemorrhage occurred in 2 patients	Full recovery (19)
3	2021	Tao Chen	China	27	30	15.0	36.0	ESD	100%	93.3%	No	emphysema occurred in 1 patients	Full recovery (20)
4	2013	Noboru Hanaoka	Japan	28	38	6.2	74.0	ESD	100%	81.6%	local recurrence was observed in 4 patients	laryngeal edema occurred in 4 patients,	Full recovery (21)
5	2012	Toshiro Iizuka	Japan	93	140	23.4	65.3	ESD under peroral countertraction	100%	69.8%	No	No	Full recovery (22)
6	2011	T Iizuka	Japan	32	42	12.5	98.0	ESD	98%	79.0%	No	postoperative subcutaneous emphysema occurred in 2 patients	Full recovery (23)
7	2006	Yuichi Shimizu	Japan	4	4	19.5	68.8	ESD	75%	50.0%	No	No	Full recovery (24)

In this study, 46.2% of cases (6/13) had positive horizontal margins. This observation may be attributed to the relatively narrow anatomical space of the hypopharynx, making it challenging to perform a wider resection during ESD. However, we performed margin marking intraoperatively. It should be noted that thermal injury to the normal marginal tissue during cautery may lead to false-positive results in pathological sections. Importantly, no recurrence was observed in the positive horizontal margin areas during the follow-up period, which further confirms the clinical effectiveness of the treatment.

In conclusion, ESD is a safe and effective treatment for superficial hypopharyngeal carcinoma, offering high resection rates, low recurrence, and preservation of normal tissue function. It is a viable alternative to traditional treatments, improving postoperative quality of life and functional outcomes.

## Data Availability

The raw data supporting the conclusions of this article will be made available by the authors, without undue reservation.
